# *Galleria melonella* as an experimental *in vivo* host model for the fish-pathogenic oomycete *Saprolegnia parasitica*

**DOI:** 10.1016/j.funbio.2017.12.011

**Published:** 2018

**Authors:** Andreas Wuensch, Franziska Trusch, Nurul A. Iberahim, Pieter van West

**Affiliations:** a Aberdeen Oomycete Laboratory, Institute of Medical Sciences, University of Aberdeen, Aberdeen, AB25 2ZD, Scotland, UK; b International Centre for Aquaculture Research and Development (ICARD), University of Aberdeen, Scotland, UK

**Keywords:** *Galleria mellonella*, Host model, Oomycetes, *Saprolegnia parasitica*

## Abstract

Oomycetes are eukaryotic pathogens infecting animals and plants. Amongst them *Saprolegnia parasitica* is a fish pathogenic oomycete causing devastating losses in the aquaculture industry. To secure fish supply, new drugs are in high demand and since fish experiments are time consuming, expensive and involve animal welfare issues the search for adequate model systems is essential. *Galleria mellonella* serves as a heterologous host model for bacterial and fungal infections. This study extends the use of *G. mellonella* for studying infections with oomycetes. Saprolegniales are highly pathogenic to the insects while in contrast, the plant pathogen *Phytophthora infestans* showed no pathogenicity. Melanisation of hyphae below the cuticle allowed direct macroscopic monitoring of disease progression. However, the melanin response is not systemic as for other pathogens but instead is very local. The mortality of the larvae is dose-dependent and can be induced by cysts or regenerating protoplasts as an alternative source of inoculation.

## Highlights

• *Galleria mellonella* serves as a heterologous host model system for Saprolegniales.
• The melanisation of the larvae is local around the growing hyphae.
• Regenerating protoplasts can be used as an alternative inoculum to cysts.

## Graphical abstract

**Figure:**
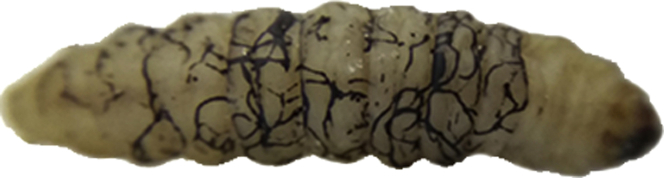

## 1 Introduction

Oomycetes are eukaryotic organisms that are morphologically similar to fungi but genetically related to brown algae (Baldauf et al., 2000). The vast majority of oomycete species are pathogenic to animals and plants, significantly affecting the agri- and aquaculture industries, but also responsible for wiping out wild populations (van West, 2006). The most devastating oomycete affecting salmon farming is *Saprolegnia parasitica* because it infects fish eggs as well as salmonid fish (Stueland et al., 2005, van West, 2006, van den Berg et al., 2013). However, also other fish in fresh water and arthropods such as crayfish and river insects are susceptible to *S. parasitica* and other Saprolegnia species (Krugner-Higby et al., 2010, Sarowar et al., 2014). The disease caused by Saprolegniaceae is called saprolgeniosis (or saprolegniasis) and once the infection is established, cotton-like white to grey patches on the skin, especially around the fins, as well as in the gills can be observed with destruction of the epidermis (Khoo, 2000, Bruno et al., 2011). While the infection progresses, fish become lethargic and lose their equilibrium (Bruno et al., 2011). Final death of the fish occurs due to an osmotic shock caused by haemodilution (Roberts, 1993, Torto-Alalibo et al., 2005).

While research on plant pathogenic oomycetes allows *in planta* infection assays without ethical objections (Kanneganti et al., 2007, Bhaskar et al., 2009), studies on fish pathogens such as *S. parasitica* on its native salmonid hosts are expensive, time consuming and raise animal welfare issues. Moreover, it is difficult to replicate the complex conditions under which infections naturally occur (Kales et al., 2007, Minor et al., 2014). Due to the economic impact of *S. parasitica*, *Saprolegnia diclina* and other *Saprolegnia* species in hatcheries, eggs are also frequently used for challenge experiments (Songe et al., 2016, Eissa et al., 2013). However, these studies aim more at replicating conditions of outbreaks on eggs specifically than investigating the host–pathogen interaction and insights of the defence system gained from egg experiments cannot simply be transferred to fish. Another way to study the immune response to saprolegniosis is the work with cell lines (Kales et al., 2007, de Bruijn et al., 2012, Belmonte et al., 2014); although the humoral immunity component is absent in cell line studies (Magnadóttir, 2006). Hence, a reliable and simple model system to screen potential compounds against Saprolegnia species and to study virulence factors under controlled conditions is needed.

Due to their low cost, easy handling, short reproductive cycles and simple housing, insects and nematodes have become increasingly popular as alternative model hosts in infection assays (Cook and McArthur, 2013). For many fungal pathogens, such as the human opportunistic pathogen *Candida albicans*, the greater wax moth (*G.* *mellonella*) is used as an alternative approach to classical mouse model system (Chamilos et al., 2007). The insects are economical, simple to maintain and viable at a wide range of temperatures allowing experimentation at temperatures more similar to the natural conditions in freshwater ecosystems (10–20 °C). *G.* *mellonella* can be used without ethical concerns and results can be obtained within days. Similar to the skin of vertebrates, the cuticle of *G. mellonella* provides the first defence barrier against pathogens (Magnadóttir, 2006). In the insect itself, the pathogen has to overcome the interconnected cellular and humoral immune response contained in the haemolymph, the blood analogue of insects (Matha & Áček, 1984; Brennan et al., 2002). Hence, the insect immune system comprises important similarities to the vertebrate immune system at a structural and functional level (Zhao and Kanost, 1996, Rock et al., 1998, Wittwer et al., 1999, Kavanagh and Reeves, 2004, Lemaitre and Hoffmann, 2007) and therefore provides a potential model system to study the pathogenicity of *S. parasitica*.

Here we report on the use of *G. mellonella* as a novel *in vivo* model to study animal pathogenic oomycetes. The virulence of two *Saprolegnia* species was determined through the mortality rates of infected insects. Progression of infection was monitored enzymatically and by visual inspection as well as histological examinations. Since the production of zoospores or cysts can be challenging, especially for newly discovered species, we show that protoplasts can also be used as an inoculum.

## 2 Material and methods

### 2.1 Galleria mellonella

Sixth instar larvae of *G. mellonella* (Lepidoptera: pyralidae) (Livefood UK Ltd. Somerset, UK) were kept in wood shavings in the dark at 12 °C. Prior to a challenge experiment, healthy insects without black or grey marks visible on the skin and a swift response to stimulation (being flipped on their backs) were separated and used. Twenty larvae for each group were inoculated with 10 μL of cysts or protoplasts or as a negative control with 10 μL PBS via the last left pro-leg into the haemolymph with a 10 μL microsyringe (VWR, 549-0199) as described elsewhere (Mylonakis et al., 2007; Bergin et al., 2006). Following inoculation, larvae were incubated at 24 °C (optimal *in vitro* growth temperature for *S. parasitica*) in the dark. Insects were investigated as indicated. An insect was scored as dead when it did not show a response to physical stimulation with forceps.

### 2.2 Oomycete strain maintenance, zoospore/cyst and protoplast production

*S. parasitica* C65 (CBS223.65) was originally isolated from young pike (*Esox lucius*), *S. parasitica* N12 (VI-02736) originated from parr of Atlantic salmon in Scotland (Lochailort) (Jiang et al., 2013). *Saprolegnia delica* (DON160516) was isolated from caddisfly larvae (*Rhyacophila dorsalis*) sampled from the river of Don near Monymusk, Scotland. *Phytophthora infestans* (P88069) was originally isolated from tomato in the Netherlands (Kamoun et al., 1998). Stock cultures of each strain were kept at 12 °C on potato-dextrose agar. For growth tests, agar plugs (⌀ 0.5 cm) were cut from the stock cultures and mycelial growth was tracked over time.

For the production of zoospores, an agar plug of mycelia was grown in pea broth (125 g pea/L) at 24 °C. Sporulation of 3 d old mycelia was induced by washing 3 times with deionised water followed by 24 h incubation in Tap:tank water (sterile water from a fish tank diluted 1:2 in tap water) at 24 °C. Zoospores were separated from sporulating mycelia through 40 μm cell strainers and encysted during the washing procedure.

For the production of protoplasts, 24 h old mycelium was digested in 0.5 M sorbitol supplemented with cellulose (5 mg/mL, Sigma–Aldrich, C8546) and lysing enzymes (5 mg/mL, from *Trichoderma harzianum*, Sigma–Aldrich, L1412) at 200 rpm at 25 °C for 1 h. Protoplasts were separated from non-degraded mycelia through 40 μm cell strainers and washed 3 times in 0.5 M sorbitol to remove digestive enzymes and small debris from the disrupted mycelia. Before injections, protoplasts were allowed to regenerate in LB medium at 24 °C for 2 h.

For injections into *G. mellonella*, cysts and protoplast were concentrated by centrifugation, counted and diluted in PBS to the final concentration as indicated.

### 2.3 Histological examination

Insect preparation for paraffin embedding was performed essentially as described elsewhere (Perdoni et al., 2014). Briefly, 50 μL of 4 % PFA/PBS was injected into the last right pro-leg before immersing insects in 4 % PFA overnight at 4 °C. Fixed insects were embedded in either paraffin or OCT embedding matrix (Cellpath, KMA-0100-00A). Samples were cut in 10 μm sections on a cryostat (Leica 1850) at −20 °C.

Staining with GMS (Grocott methamine-silver) was performed by the NHS Grampian Biorepository (Aberdeen, Scotland). Briefly, slides were deparaffinised, hydrated and oxidised in 2 % chromic acid for 5 min. Treatments in 1 % sodium metabisulfit (1 min), quickly in methenamine silver solution and 0.5 % gold chloride followed before a final incubation in 5 % sodium thiosulfate (3 min). Counterstaining was performed with working light green solution. Slides were thoroughly rinsed with water between individual steps.

For PAS stain slides were deparaffinised, hydrated and oxidised in 0.5 % periodic acid schiff (PAS) for 5 min. Followed by an incubation in Schiff reagent for 15 min and a counter stain with Mayer's Haematoxylin Solution for 1 min. Slides were thoroughly rinsed with water between individual steps. Slides were scanned on a Zeiss Axio Scan Z1.

For whole mount microscopy insects were opened and the cuticle spread out on a glass slide after removing internal organs. Samples were inspected on an inverted microscope and images were taken using a Lumenera Infinity2 camera.

### 2.4 DNA extraction

For PCR amplification, DNA was isolated from mycelia and infected larvae of *G. mellonella*. A piece of pea-broth-cultured mycelium or a cut larvae were transferred into 2 mL tubes with glass beads (425–600 μm) and 800 μL DNA extraction buffer (Sarowar et al., 2014) containing RNAseA and homogenized in a FastPrep-24™ 5G tissue homogenizer (MP Biomedicals, 4 × 40 s, 6 m/s, 2 min breaks). After disruption samples were incubated at 55 °C for 30 min followed by a centrifugation to remove debris at 10,000 × g for 10 min. The supernatant was used for DNA extraction by a phenol-chloroform method as described elsewhere (Zelaya-Molina et al., 2011). Briefly, for DNA extraction from insects an additional phenol extraction step was performed. DNA was precipitated in 1 mL isopropanol overnight at −20 °C and pelleted by centrifugation at 4 °C. Pellets were washed twice with 70 % ethanol before air dried at room temperature for 5 min, followed by resuspension in nuclease-free water.

### 2.5 PCR

Standard PCR for ITS amplification was performed with the GoTaq polymerase according to manufacturer's instructions (Promega) with 0.2 μM primer each (ITS4 (5′- TCCTCCGCTTATTGATATGC-3′) and ITS5 (5′-GGAAGTAAAGTCGTAACAAGG-3′)) with T_m_ = 58 °C and an elongation time of 1 min.

### 2.6 Phenoloxidase activity

For measuring the phenoloxidase (PO) activity challenged larvae were incubated on ice for 10 min. 10 μL haemolymph were collected from the tail between the prolegs and diluted with 25 μL PBS. Cells were pelleted (5 min, 500 × g, 4 °C) and 10 μL of the supernatant was added to 200 μL l-DOPA (10 mM). To allow substrate conversion samples were incubated for 1 h at 30 °C. Absorption of melanin was measured at 490 nm.

## 3 Results

### 3.1 Injection of cysts of *S. parasitica* strains results in killing and colonization of *G. mellonella* larvae

**Fig. 1:**
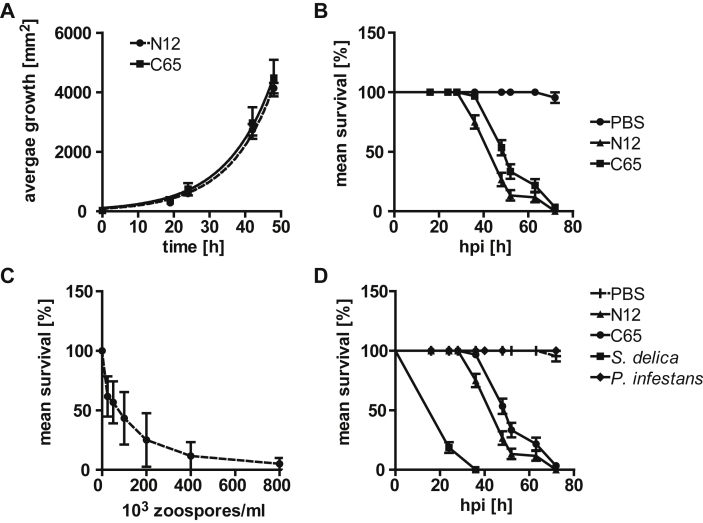
Mortality of *G. mellonella* after injection with cysts of different oomycete isolates. (A) *S. parasitica* strains N12 (dashed line) and C65 (solid line) show the same *in vitro* growth rate on agar plates. (B) *G. mellonella* was injected with 100 cysts of either N12 (▲) or C65 (■). Infections with N12 progress faster compared to C65 while the cumulative mortality rate after 3 dpi is the same. (C) The survival rate of *G. mellonella* at 48 hpi decreases with an increasing concentration of injected cysts of *S. parasitica* N12. (D) Comparison of *S. parasitica* C65 (●) with the insect pathogenic *S.delica* (■) and the plant pathogenic *P.infestans* (◆). While *S. delica* causes a higher mortality rate, G*. mellonella* is not susceptible to cysts of *P. infestans* even after increasing the amount of injected cysts (Fig. S1).

Cysts of *S. parasitica* were collected and injected into the haemocoel of healthy larvae. For comparison, the reference strain C65 and N12, of which the latter one is significantly more virulent in Atlantic salmon, were used (Jiang et al., 2013). Although both strains show the same *in vitro* growth rate on agar plates (Fig. 1A), their virulence to moth larvae differs. Consistent with a higher pathogenicity of N12 in Atlantic Salmon, N12 resulted in a higher mortality rate of *Galleria melonella* compared to C65 (Fig. 1B). During the first 24 hpi no mortalities were observed, while on day 2 both strains caused rapid mortality. Although both strains were able to cause almost 100 % cumulative mortality within 72 h (day 3), infections with N12 were more progressive than with C65 (75 % and 50 % mortality after 48 h, respectively). As expected, the progression of the infection process is faster when more cysts are injected (Fig. 1C). When injecting germinated cysts from the insect pathogenic oomycete *S.* *delica* into *G. mellonella*, mortality rates were increased and killing occurred even within 24 hpi (Fig. 1D). In contrast, cysts of the plant pathogenic oomycete *P.* *infestans* were unable to kill larvae, even when the concentration of cysts was increased to 2450 per insect (Fig. S1). In none of the challenges a transmission from dead insects to healthy larvae could be observed. Mycelia could only be re-isolated from oomycete- but not from PBS-treated insects.

### 3.2 Phenotype of *Saprolegnia* infection in *G. mellonella*

**Fig. 2:**
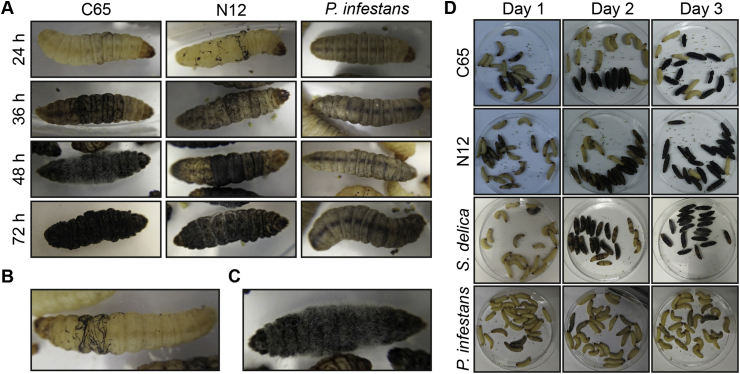
Progress of infection with *Saprolegnia* in *G. mellonella*. (A) Disease progression is associated with increasing melanisation over time. First signs of infections caused by *S. parasitica* are localised pigmentations around the mycelium. Later the entire insect turns dark until death. While *S. parasitica* N12 infected insects keep their shape, *S. parasitica* C65 penetrates the cuticle and insects shrink. Infection with *P. infestans* only causes a weak systemic melanisation of larvae that does not change over the time. (B) Local melanisation of the thorax of an *S. parasitica* C65 infected *G. mellonella* at early stages. (C) Penetration of the cuticle by *S. parasitica* C65 after the insect died. The penetration leads to water evaporation and concomitant shrinking of the body. (D) Progress of *Saprolegnia* infection over time after infection of *G. mellonella* with cysts of *S. parasitica* C65, N12 and *S. delica.* After infection with *P. infestants* early systemic melanisation appears only in some insects and shows little progression.

A key part of the humoral immune response of *G. mellonella* comprises the formation of brown-coloured melanin by phenoloxidases to encapsulate invading pathogens and thereby preventing their growth by inducing starvation (Gillespie et al., 1997, Cerenius and Söderhäll, 2004, Eleftherianos and Revenis, 2011, González-Santoyo and Córdoba-Aguilar, 2012). Due to the obvious difference between yellowish healthy larvae and the melanin induced brown colour of infected insects, the progression of an infection can be followed macroscopically (Fig. 2). When comparing larvae infected with *S. parasitica* strains C65 or N12, the stages of infection are very similar (Fig. 2A). Up to 24 hpi the insects are generally asymptomatic with a high vitality as no signs of infection can be seen and the response to physical stimuli is unchanged. The first visible signs of infection appear when black lines in the abdomen and thorax are formed. These are attempts from the larvae to encapsulate the mycelium by local melanisation (Fig. 2B). If the network of mycelium becomes visible underneath the cuticle, the corresponding insect will die within the next 24 h. As the infection progresses, black patches are expanding and melanisation is detected at additional sites. On day 2, the whole insect begins to turn dark while the mycelium expands throughout the whole larvae and the first mortalities occur. Until death of *G. mellonella* clinical signs for both *S. parasitica* strains are the same. However, for a short time after death, mycelium of C65 penetrates through the cuticle from the inside of the insect and is exposed to the environment (Fig. 2C). Shortly after, the dead insects begin to shrink and shrivel. In contrast, the cuticle of insects infected with N12 is not perforated by mycelium (Fig. 2A). Within 72 hpi the cumulative mortalities peak for N12 as well as C65 and the insects that do not yet show any signs of infection tend to survive and keep their yellowish appearance (Fig. 2D). In contrast, dead insects are entirely black after 3 d. The same phenotype can be observed also with other Saprolegniales (Fig. 2D). However, after injection with cysts of *P. infestans*, a faint melanisation of the whole larvae is observed that does not change over time while the mortality rate is very low (Fig. 2D).

### 3.3 Histopathology of *G. mellonella* suffering from infection with *Saprolegnia*

**Fig. 3:**
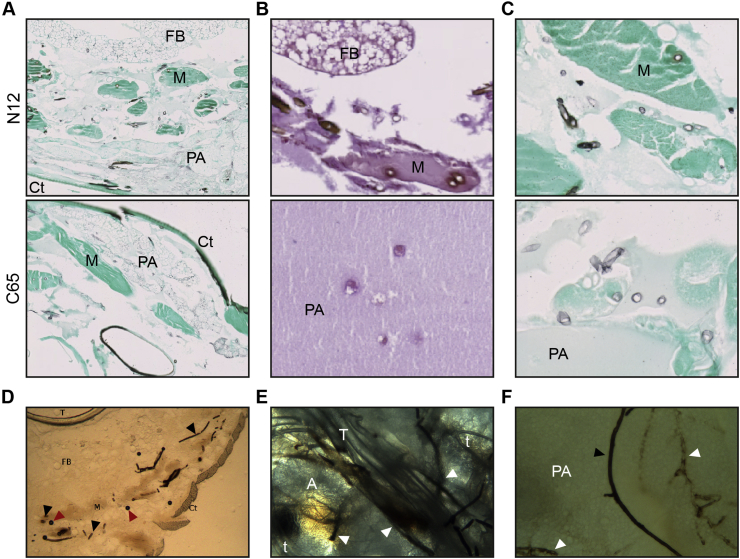
Histopathology of *Saprolegnia* infection in *G. mellonella*. (A) GMS stain of tissues of infected insects with *S. parasitica* N12 and C65. Mycelium appears as black lined circles (black arrows) which cannot be detected in the control (Fig. S2A). Both strains cluster at the musculature (M) and the trachea (T), spreading into the peripheral adipose tissue (PA) below the cuticle (Ct). The gut associated fat-body (FB) was mostly free of mycelia. (B) In PAS stained samples, *S. parasitica* N12 and C65 appear dark pink with brown halos when the mycelium is melanised. (C) Beside the same localisation in the musculature, N12 mycelium is stronger and more frequently melanised compared to C65. (D) The high melanisation of *S. parasitica* N12 allows the detection of mycelium in unstained cryo-sections of infected insects (black arrow heads). Tracheoles (red arrow heads) have a larger diameter than mycelium. M: muscle, Ct: cuticle, FB: fat-body, T: trachea. (E) Whole mount preparation of a life insect showing clinical signs after infection with *S. parasitica*. Mycelium (arrow heads) is easier to distinguish from trachea (T) or tracheoles (t) compared to cryo-sections. (F) Peripheral adipose (PA) tissue from a whole mount preparation including one fully (black arrow head) and partially melanised hyphae (white arrow heads).

In order to study changes in the insect tissue, histological sections from *S. parasitica* infected insects were microscopically examined. C65 as well as N12 were both found in subcuticular tissues (including the peripheral fat body), trachea and particularly in muscle but was absent in the gut-associated/perivisceral fat-body (Fig. 3A). Hyphae of *S. parasitica* C65 as well as N12 were GMS positive while in the PBS-injected control group no hyphal structures could be detected (Fig. S2A). This was also confirmed by the Periodic acid Schiff (PAS) stain in which mycelium appears as dark pink (Fig. 3B). On average hyphal structures of C65 and N12 have a diameter of 6 μm and 7.5 μm, respectively, which is very similar to the tracheoles of *G. mellonella*. However, while hyphal structures are very consistent in diameter, tracheoles differ because of their narrowing tips and in addition, they are GMS negative. As a reaction of the insect immune system to the infection and as already seen macroscopically, melanisation also appears microscopically as brown halos around GMS stained mycelium (Fig. 3C). Although the phenotypes in *G. mellonella* caused by an *S. parasitica* C65 and N12 infection are very similar (Fig. 2A), N12 mycelium is more frequently and more intensely melanised than C65 is (Fig. 3C). Due to the high melanisation of hyphae of *S. parasitica* it is possible to observe mycelia without any stain of sections (Fig. 3D) as well as whole-mount unprocessed tissue (Fig. 3E). The whole-mount processing is very time efficient and allows for easy distinguishing between hyphae and trachea since they differ in colour, size and shape. Furthermore, the examination of unstained samples confirms a gradual melanisation process since partially melanised hyphae can be detected at various sites (Fig. 3F).

### 3.4 Detection of *S. parasitica* in challenged *G. mellonella*

The activity of melanin-producing phenoloxidases (PO) in the haemolymph can also be measured biochemically *in vitro* and is used for the determination of the systemic immune response in *G. mellonella* (Dubovskiy et al., 2013). Although the level of melanisation is different for *S. parasitica* strains N12 and C65, the PO activity in the haemolymph is identical (Fig. S2B). However, 24 hpi the activity is below the PO activity measured in PBS-injected samples. Therefore, this assay is to be considered as not suitable for the detection of an infection with an oomycete.

**Fig. 4:**
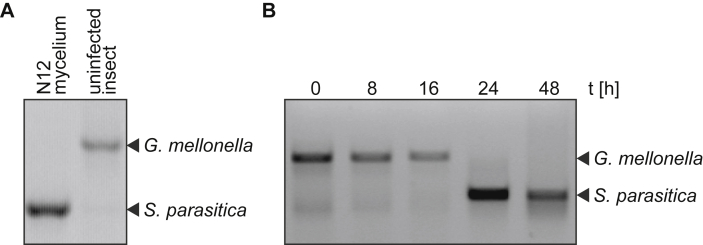
Detection of *S. parasitica* inside infected *G. mellonella*. (A) PCR reaction (ITS-4/5) of a pure culture of *S. parasitica* N12 resulted in a band of 800 bp while for non-infected insects a band of 1500 bp appears. (B) Time course of the infection progress of *G. mellonella* infected with *S. parasitica*. PCR was run on whole sample DNA extractions. As the infection proceeds, the ITS-4/5 primer favour the *S. parasitica* product in challenged insects after 24 h while the band of *G. mellonella* gradually disappears. The 800 bp fragment from infected insects was sequenced and matched to the ITS region of *S. parasitica*.

In order to confirm results of macroscopic and microscopic examinations of the *Saprolegnia* infections, PCR was used to detect also minimal amounts of *S. parasitica* DNA. Primers covering the high copy number ITS (internal transcribed spacer) region were used for DNA amplification. Amplification from a pure oomycete strain resulted in the usual size of 800 bp for the ITS region while in non-challenged insects the ITS region (1.5 kb) of *G. mellonella* was amplified (Fig. 4A). Hence, as infections progress the intensity of the *G. mellonella* band decreases and the *S. parasitica* band appears (Fig. 4B).

### 3.5 *G. mellonella* infection with protoplasts of *S. parasitica*

**Fig. 5:**
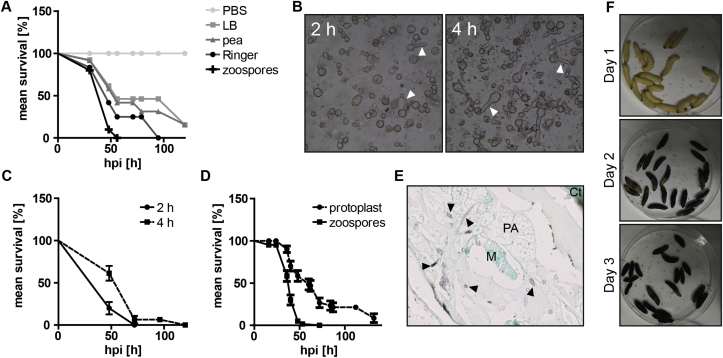
Protoplast as an alternative inoculum for *S. parasitica*. (A) Effect of regeneration medium on protoplast virulence. Prior injections, protoplast were regenerated in different buffers as indicated. Mortality of *G. mellonella* after injection with 200 protoplast of *S. parasitica* C65 was measured over time. (B) Protoplast of *S. parasitica* C65 recovering and germ tube formation (arrow heads) is progressed after 4 h compared to 2 h. (C) Effect of regeneration time on the virulence of protoplasts. The virulence of regenerated protoplast is stronger after 2 h (●) compared to 4 h (■). (D) Cumulative mortality of *G. mellonella* 48 hpi is indistinguishable between infections with protoplast (●) or cysts (■) of *S. parasitica* C65. (E) Histological examination of *G. mellonella* infected with *S. parasitica* protoplasts revealed the same localisation of mycelium (arrow heads) compared to infections with cysts. M: muscle, Ct: cuticle, PA: peripheral adipose tissue, T: trachea. (F) Progress of *Saprolegnia* infection in *G. mellonella* over time after inoculation of protoplasts is the same compared to cysts-injected insects (Fig. 2D).

Cysts are the natural vehicle of most oomycete infections. However, spore release can be unreliable in terms of numbers of cells and the time of release, which complicates the performance of infection challenges with *G. mellonella*. Therefore, protoplast injections were explored as an alternative source of infection. The protoplast regeneration buffer used was found to be a critical parameter for successful protoplast recovery rates and thereby for infectiousness (Fig. 5A). In addition, regeneration of protoplasts before infection is beneficial for the infection process of *G. mellonella* but only up to a particular length of the germ tubes (Fig. 5B and C). Finally, after optimisation of the regeneration medium and time, mortality rates of *G. mellonella* challenged with either protoplast or cysts of *S. parasitica* C65 were comparable (Fig. 5D). Additionally, infections caused by protoplasts were indistinguishable from cysts infections in disease progression and during histological examinations (Fig. 5E and F). Although protoplasts are not a natural source of infection, they can be a viable alternative to cysts in order to study oomycete infections in *G. mellonella*.

## 4 Discussion

After injection, cysts or protoplasts from *S. parasitica* are able to establish an infection in the alternative heterologous host *G. mellonella*. The resulting clinical symptoms can be used to distinguish between different strains of *S. parasitica*. The susceptibility of *G. mellonella* seems to be restricted to animal pathogenic oomycetes because after infection with *P. infestans* we did not observe significant mortalities.

Compared to small aquatic organisms like river insects, *G. mellonella* does not have to be captured for inspection and inoculation is more convenient because of the size of the insects. In addition, disease progression is more isolated and free of external influences. Furthermore, compared to *Drosophila melanogaster*, *G. mellonella* is susceptible to a broad range of pathogens and no immune-deficient mutants have to be used (Brennan et al., 2002, Perdoni et al., 2014). The zoospore/cysts production in oomycetes for infection experiments can be quite challenging and a final working protocol requires a lot of optimisation for newly discovered strains. After regeneration, protoplast share many morphological similarities with germinated cysts in our study. Indeed, injections with protoplasts replicated infections with cysts indistinguishably and therefore provide a reliable alternative as infection agents (Butt et al., 1996).

In previous reports, *S. parasitica* N12 was found to be one of the most virulent cultured strains (Stueland et al., 2005) which is in agreement with the slightly higher mortality of *G. mellonella* compared to infection with *S. parasictica* C65 in our study. Challenges of *G. mellonella* with a higher inoculum of the human pathogenic *C. albicans*, *Staphylococcus aureus*, *Shigella* spp. or *Aspergillus fumigatus*, resulted in earlier clinical signs but a lower overall mortality rate compared to infections with the Saprolegniales tested in this study (Desbois and Coote, 2011, Li et al., 2013, Barnoy et al., 2017, Mulvihill et al., 2017). Furthermore, the infection of *G. mellonella* with animal pathogenic oomycetes (*S. parasitica* and *S. delica*) is more efficient than with the plant pathogenic oomycete, *P. infestans*. While shrunken carcasses of insects challenged with *S. parasitica* C65 become hard and dry, presumably due to water evaporation through the perforated cuticle, insects injected with cysts from *P. infestans* show reduced response rates and no significant mortality. It was possible to detect melanised mycelia in surviving insects (Fig. S3) which might indicate the capability of *G. mellonella* to cope with a mild infection with *P. infestans*.

Although melanisation is a key part of the immune system of the insect, no PO activity could be detected in the haemolymph from *G. mellonella* infected with *S. parasitica* (Fig. S2B) which is in contrast to studies with other fungi and bacteria where PO activity was detected (Harding et al., 2012, Grizanova et al., 2014, Kryukov et al., 2016). However, in contrast to infections with human fungi, the immune response to *S. parasitica* is very local around melanised hyphae and is not systemic. Thus, it is likely that initially only a small amount of POs is activated locally when haemocytes are locally committed and found less in the circulation (Lavine and Strand, 2002). Hence, the PO activity assay is an unsuitable tool to measure the immune response to oomycete infections.

Although the insect's immune system is mainly limited to an innate defence response (Hillyer, 2016), *G. mellonella* is still a reasonable model system for infections with *Saprolegnia* since it has been shown that *S. parasitica* is mainly facing the innate immunity of fish because the adaptive immune system is suppressed and the progression of infection is often too fast for an immune response (Belmonte et al., 2014). The first macroscopic sign of the humoral immune response is the melanisation and thereby encapsulation of subcuticular hyphae (Cerenius and Söderhäll, 2004, Eleftherianos and Revenis, 2011, González-Santoyo and Córdoba-Aguilar, 2012), which is considered as a point of no return because in our studies none of the larvae were able to recover from the infection. At later stages of infection also the haemolymph becomes pigmented, indicating a systemic response. Interestingly, although *S. parasitica* N12 is slightly more pathogenic, the melanisation response is stronger and thereby likely causing a stronger immune response when compared to injections with C65. Melanin is produced by phenoloxidases (PO) which are expressed as inactive zymogens (Cerenius and Söderhäll, 2004). POs are not secreted but released by the rupture of haemocytes into the extracellular space where they are activated by for example components of the pathogen's cell wall (Cerenius and Söderhäll, 2004, Kanost and Gorman, 2008). However, the activity of POs does not always correlate with resistance to an infection since in some cases an excessive response can lead to the death of the insect (González-Santoyo and Córdoba-Aguilar, 2012). Nonetheless, reports indicate a positive correlation of virulence in *G. mellonella* and mammals (Jander et al., 2000, Brennan et al., 2002). Understanding of the melanin production in *G. mellonella* in response to Saprolegniales might also increase the understanding of infections in crayfish, which immune system also comprises melanisation to combat infections (Noonin et al., 2010).

Histological examinations revealed the spread of mycelium occurs segmentally and primarily along sub-cuticular regions close to muscles and from there into adjacent tissues with the least mycelium in tissues most distal from the site of infection. Interestingly, even in highly infected insects hardly any mycelium was detected in the gut-associated/perivisceral fat body which is the main source for the production of antimicrobial peptides in *G. mellonella* (Arrese and Soulages, 2010).

In this study, *G. mellonella* was established as an alternative model host to study the virulence of animal pathogenic oomycetes *in vivo*. *G.* *mellonella* offers a low cost, fast replicative model systems without ethical concerns that allow screening experiments before performing animal (fish) experiments.

## Appendix A Supplementary data

mmc1
