# Supplementary material for: Galleria melonella as an experimental in vivo host model for the fish-pathogenic oomycete Saprolegnia parasitica
Source: Fungal Biol. 2018 Feb-Mar;122(2-3):182–9. doi: 10.1016/j.funbio.2017.12.011 (PMC5840505; doi:10.1016/j.funbio.2017.12.011)
Supplement: mmc1 [file mmc1.docx]

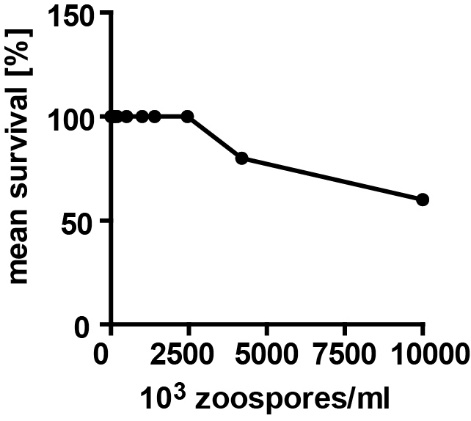


Figure S1: Mortality of *G. mellonella* after injection with zoospores of *P. infestans*.

*G. mellonella* was injected with different concentrations of zoospores of *P. infestans* as indicated. In contrast, to injections with *S. parasitica* (Fig. 1C), *P. infestans* shows only moderate mortality at 1000 times higher concentration of cyst after 5 days.


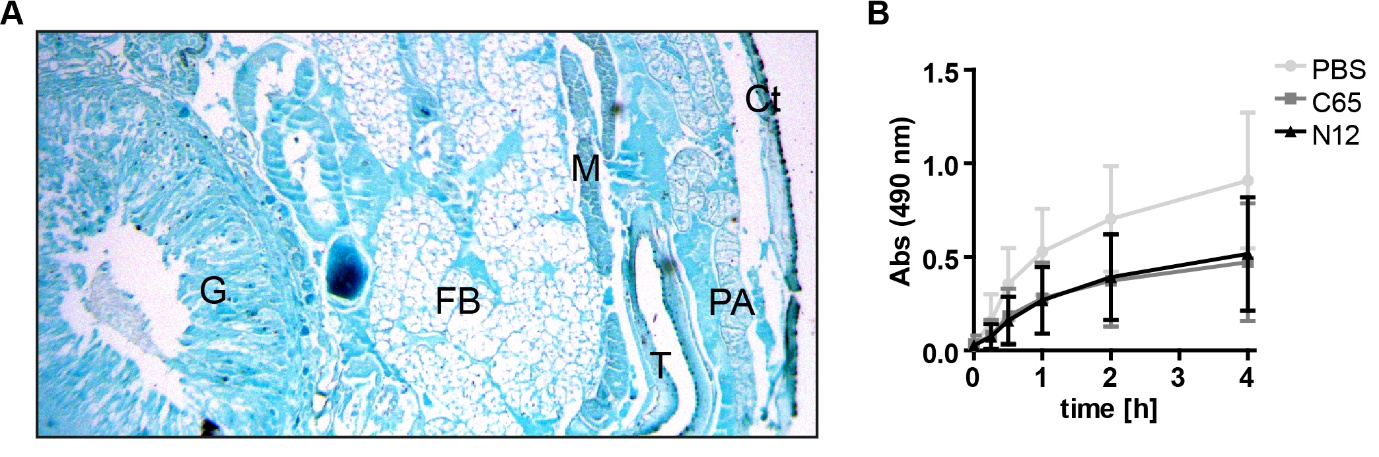


Figure S2: Melanisation of hyphae.

(A) GMS stain of tissues of non-infected insects. Black mycelium can only be detected in infected larvae (Fig. 3A-C). M: musculature, T: trachea, PA: peripheral adipose tissue, Ct: cuticle, FB: gut associated fat-body, G: midgut. (B) Analysis of PO activity in the haemolymph from *G. mellonella* larvae infected with *S. parasitica* C65 and N12. L-dopamine substrate solution was added, and absorbance at 490 nm was monitored. The PO activity in the haemolymph of *S. parasitica* infected insects is less than in the control group with PBS injected larvae.


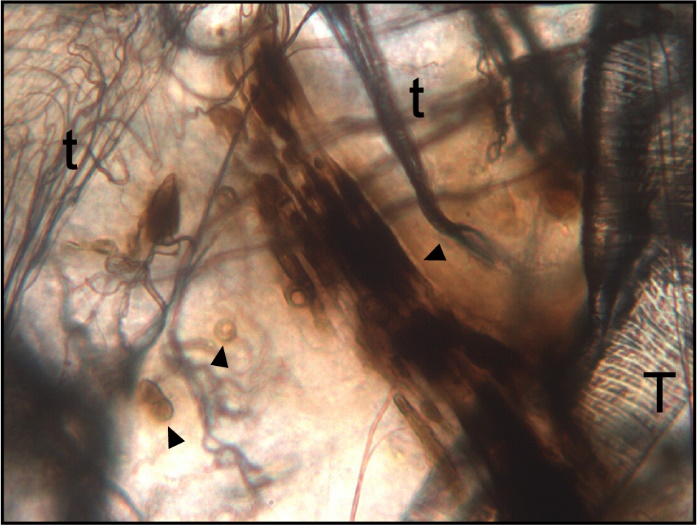


Figure S3: In situ detection of *P. infestants* in *G. mellonella*.

Whole mount preparation of a life insect showing clinical signs after infection with *P. infestans*. Although no melanised hyphae are seen from the outside (Fig. 2A), cluster of hyphae can be observed inside an insect challenged with 10,000 zoospores. Melanised Mycelium (arrow heads), T: trachea, t: tracheoles.
